# Mechanical power and normalized mechanical power in pediatric acute respiratory distress syndrome

**DOI:** 10.3389/fped.2024.1293639

**Published:** 2024-01-16

**Authors:** Farhan A. R. Shaikh, Karthik N. Ramaswamy, Dinesh K. Chirla, Shekhar T. Venkataraman, Martin C. J. Kneyber

**Affiliations:** ^1^ Department of Pediatric Intensive Care, Rainbow Children’s Hospital, Hyderabad, India; ^2^ Department of Pediatric Intensive Care, Rainbow Children’s Hospital, Chennai, India; ^3^ Departments of Critical Care Medicine and Pediatrics, University of Pittsburgh School of Medicine, Pittsburgh, PA, United States; ^4^ Division of Paediatric Critical Care Medicine, Department of Paediatrics, Beatrix Children’s Hospital, University Medical Center Groningen, University of Groningen, Groningen, Netherlands; ^5^ Critical Care, Anaesthesiology, Peri-Operative & Emergency Medicine (CAPE), University of Groningen, Groningen, Netherlands

**Keywords:** pediatric acute respiratory distress syndrome, mechanical power, normalized mechanical power, mechanical energy, mechanical ventilation, ventilator-induced lung injury

## Abstract

**Background:**

Mechanical power (MP) refers to the energy transmitted over time to the respiratory system and serves as a unifying determinant of ventilator-induced lung injury. MP normalization is required to account for developmental changes in children. We sought to examine the relationship between mechanical energy (ME_BW_), MP normalized to body weight (MP_BW_), and MP normalized to respiratory compliance (MP_CRS_) concerning the severity and outcomes of pediatric acute respiratory distress syndrome (pARDS).

**Method:**

In this retrospective study, children aged 1 month to 18 years diagnosed with pARDS who underwent pressure-control ventilation for at least 24 h between January 2017 and September 2020 were enrolled. We calculated MP using Becher's equation. Multivariable logistic regression analysis adjusted for age, pediatric organ dysfunction score, and oxygenation index (OI) was performed to determine the independent association of MP and its derivatives 24 h after diagnosing pARDS with 28-day mortality. The association was also studied for 28 ventilator-free days (VFD-28) and the severity of pARDS in terms of OI.

**Results:**

Out of 246 admitted with pARDS, 185 were eligible, with an overall mortality of 43.7%. Non-survivors exhibited higher severity of illness, as evidenced by higher values of MP, MP_BW_, and ME_BW_. Multivariable logistic regression analysis showed that only ME_BW_ but not MP, MP_BW_, or MP_CRS_ at 24 h was independently associated with mortality [adjusted OR: 1.072 (1.002–1.147), *p* = 0.044]. However, after adjusting for the type of pARDS, ME_BW_ was not independently associated with mortality [adjusted OR: 1.061 (0.992–1.136), *p* = 0.085]. After adjusting for malnutrition, only MP at 24 h was found to be independently associated. Only MP_CRS_ at 1–4 and 24 h but not MP, MP_BW_, or ME_BW_ at 24 h of diagnosing pARDS was significantly correlated with VFD-28.

**Conclusions:**

Normalization of MP is better related to outcomes and severity of pARDS than non-normalized MP. Malnutrition can be a significant confounding factor in resource-limited settings.

## Introduction

Mechanical power (MP) is a composite metric representative of the energy delivered to the lung and unifies the determinants of ventilator-induced lung injury (VILI) (1–3). MP includes elastic, static, and dynamic components, such as driving pressure (Δ*P*), positive end-expiratory pressure (PEEP), and “non-pressure” parameters such as respiratory rate (RR), inspiratory flow (F), inspiratory time (Ti), and tidal volume (Vt), all found to be independently associated with VILI (1–9). MP is associated with higher mortality in adults with acute respiratory distress syndrome (ARDS) (3, 10). Through a secondary analysis of the Pediatric Acute Respiratory Distress Syndrome Incidence and Epidemiology (PARDIE) study data, it was reported that high MP was associated with fewer 28-day ventilator-free days (VFD-28), especially among children <2 years of age (11).

The concept of MP was proposed from data in the adult population who were ventilated in volume-controlled mode, which is characterised by a constant inspiratory flow and a zero-flow state to measure plateau pressure (Pplat) initially limiting rapid implementation in paediatric critical care as children are more commonly ventilated in pressure-controlled modes of ventilation (12). This is no longer a problem as several proposed simplified equations for MP now allow calculation with these ventilation modes (13). Apart from this, several other limitations need to be addressed. In children, as the age increases, Vt increases and RR decreases, thereby inherently making it challenging to interpret MP estimates (12). Kneyber et al. proposed a modification called mechanical energy (ME_BW_) by normalizing Vt to body weight and removing RR from the equation of MP (12). They found that ME_BW_ did not change with age and correlated better with VFD-28 than MP (12). Eliminating RR from the equation may not be a good solution, as one group of investigators found that RR remained a significant outcome predictor in severe pediatric ARDS (pARDS) (14). Coppola et al. suggested normalizing MP to respiratory system compliance (MP_CRS_) and found that it is associated with adult ARDS mortality (15). Normalizing MP to respiratory compliance (C_RS_) is a tool yet to be studied in the pediatric population. Therefore, we sought to primarily investigate the association between MP, MP normalized to body weight (MP_BW_), and MP_CRS_ and mortality in children with pARDS according to the 2015 Pediatric Acute Lung Injury Consensus Conference (PALICC) definition (16). We hypothesized that normalized MP correlates better with mortality and other outcome variables (VDF-28) than MP.

## Materials and methods

We conducted a retrospective analysis of data from children with pARDS, aged 1 month to 18 years, admitted between January 2017 and September 2020 to a 24-bedded quaternary-care pediatric intensive care unit (PICU). The inclusion criteria were pediatric patients ventilated for more than 24 h, with complete medical record data. The exclusion criteria were high-frequency oscillatory ventilation (HFOV) or extracorporeal membrane oxygenation (ECMO) within 24 h of diagnosis of pARDS, patients only managed with non-invasive ventilation, and patients with neuromuscular disorders or primary immunodeficiency. pARDS was diagnosed per the PALICC definition of 2015 (16). The Hospital Ethics Committee Board, registered under the Department of Health and Research (File No. EC/NEW/INST/2021/1536), waived the need for informed consent (Letter No. RCHBH/193/12-2020). Data collected included demographic, clinical, and ventilatory parameters (Supplementary Material).

All children with pARDS were ventilated in the pressure control (PC) mode at a Vt of 6–8 ml/kg to keep the Pplat below 28 cmH_2_O, and in cases where there was evidence of poor chest wall compliance, efforts were made to keep the Pplat below 30–32 cmH_2_O as per the PALICC recommendations (16). All children were well sedated and even paralyzed if needed to eliminate spontaneous breathing and patient-ventilator asynchrony during the first 24–48 h. As per our unit policy, the nurses note the ventilator parameters in the ventilator chart at 1-h intervals. The inspiratory time on the ventilator in our unit is always set long enough to allow the inspiratory flow to touch the baseline. We collected the data at 1–4 h of inclusion in the study and after 24 h in the form of an average of two consecutive readings from the ventilator chart to calculate mechanical power and other parameters. We used measured body weights at the time of their admission to the PICU instead of ideal body weight based on height due to the high prevalence of malnutrition in our study population as per Gomez classification (17). All patients were ventilated using the guidelines described in the Supplementary Material. We used the Pediatric Risk of Mortality III (PRISM-III) score to assess the severity of illness in the first 24 h (18). The pediatric Sequential Organ Failure Assessment (pSOFA) score was used to assess the degree of severity of multi-organ failure (19).

### Power and energy calculations

MP was calculated using the surrogate formula suggested for PC mode – [MP (Joule/min) = 0.098×RR × VT × (Δ*P* + PEEP)], where Δ*P* is the difference between peak inspiratory pressure (PIP) and PEEP (13, 20). We used expiratory Vt for our calculations. MP_BW_ was calculated by MP divided by the actual body weight. We calculated the C_RS_ by dividing Vt by the difference between PIP and the PEEP, and calculated the MP_CRS_ by dividing MP by the C_RS_. We also calculated the ME_BW_ by removing RR from the surrogate PC formula and normalized it to body weight (1, 2, 10).

### Outcome measures

#### Primary outcome

To study the association between MP, ME_BW_, MP_BW_, and MP_CRS_ at 24 h of diagnosing pARDS and 28-day mortality in children with pARDS.

#### Secondary outcome

To study the association of normalized MP, MP_BW_, ME_BW_, and MP_CRS_ at 24 h with VDF-28 and oxygenation index (OI), in children with pARDS.

The 28-day mortality was defined as mortality of invasively ventilated children within 28 days of being diagnosed with pARDS. VFD-28 was defined as the number of days of unassisted breathing until day 28 of recruitment in the study, assuming a child survives for at least 2 consecutive days after initiation of unassisted breathing and remains free of assisted breathing. For patients who died within 28 days, 28-day VFD is considered zero.

### Statistical analysis

Continuous variables are expressed as medians with interquartile ranges (IQRs), and categorical variables are expressed as total numbers and percentages. Comparisons between survivors and non-survivors were made using the Wilcoxon rank-sum test for continuous variables and the chi-squared or Fischer exact test for categorical variables. The Kruskal–Wallis test was used to compare the continuous distribution of MP, MP_BW_, MP_CRS_, and ME_BW_ in mild, moderate, and severe ARDS groups. Specific sample pairs within severity subgroups of pARDS were analyzed using the Mann–Whitney *U*-test. Correlation analysis was performed between MP, MP_BW_, MP_CRS_, and ME_BW_ with OI and 28-day VFD to obtain Spearman's rank correlation coefficient (rho). Logistic regression models were created for MP, MP_BW_, MP_CRS_, and ME_BW_ at 24 h as single covariables using the enter method, with mortality as a dependent variable adjusting for clinically significant confounders like age, male gender, type of pARDS (pulmonary or non-pulmonary pARDS), presence of malnutrition, pSOFA at 24 h, and OI at 24 h. Multicollinearity diagnostics were done to detect overlapping predictors in the model by assessing for the variance inflation factor. Receiver operative characteristic (ROC) analysis was done for MP, MP_BW_, MP_CRS_, and ME_BW_ with 28-day mortality. A *p*-value <0.05 was considered significant. The statistical analysis was conducted using IBM SPSS statistics for Mac, Version 23.0 (IBM SPSS Inc., Chicago, IL, USA).

## Results

**Figure 1:**
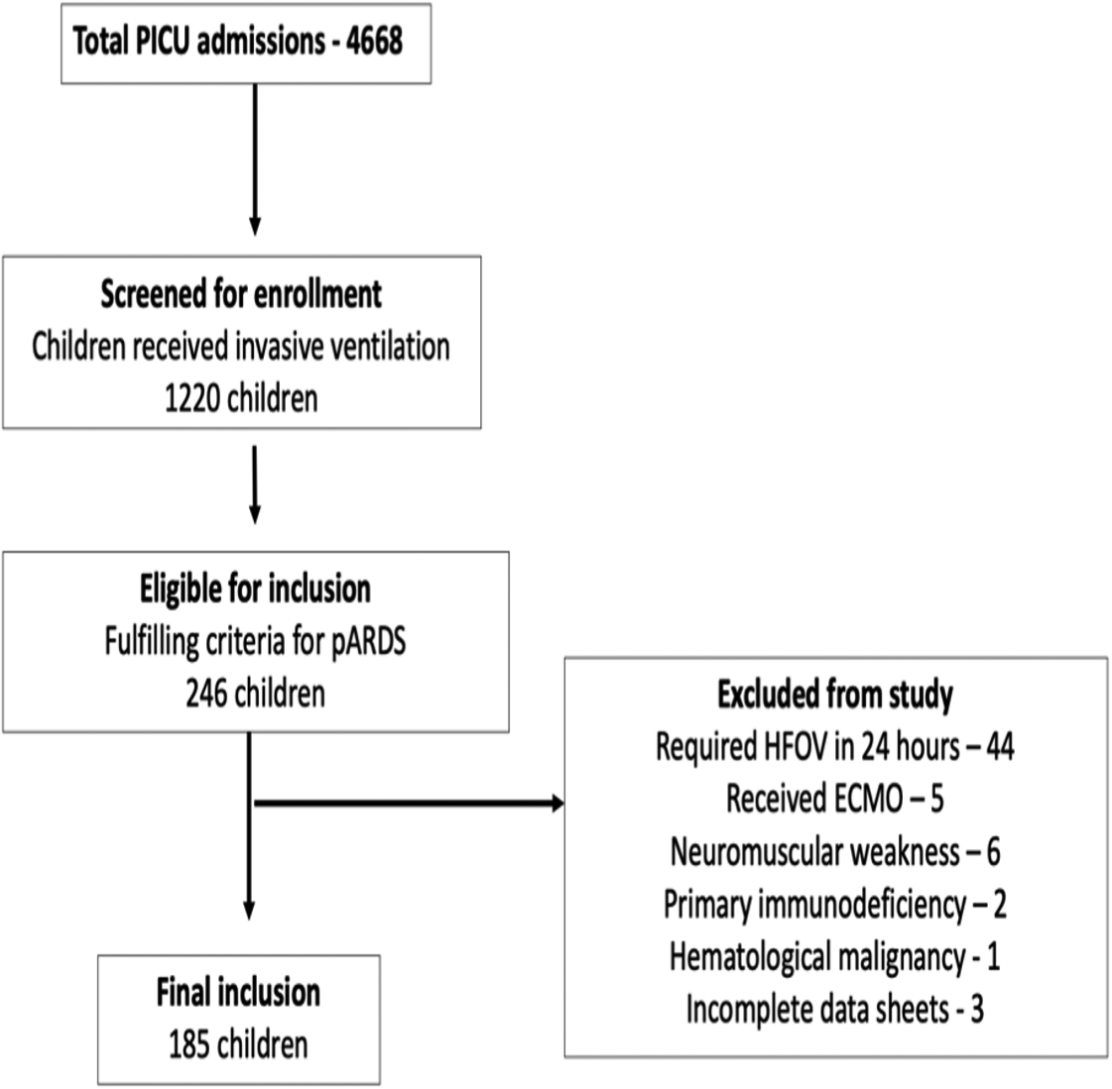
Study flow.

During the study period, 4,668 children were admitted, of whom 1,220 were invasively ventilated. Two hundred and forty-six (20.2%) met pARDS criteria, of whom data from 185 patients (75.2%) were complete and eligible for analysis (Figure 1). The median age was 1.3 (0.4–5.0) years; most patients were male (58.3%). Fifty-nine children (31.9%) were malnourished, as defined by the World Health Organization classification (17). PRISM-III and pSOFA at 1–4 h of diagnosing pARDS had medians of 8 and 8, respectively (Supplementary Table S1). At 1–4 h, the median PF ratio was 149 (116–185), and the OI was 8.3 (6.3–12.3). Among the 185 patients, 89 (48.1%), 76 (41.0%), and 20 (10.8%) had mild, moderate, and severe pARDS, respectively. The distribution of baseline characteristics by severity of pARDS is given in Supplementary Table S1. The overall mortality in the cohort was 43.7% (*n* = 81/185), with the highest mortality (70%) reported for patients with severe pARDS (Supplementary Table S2). Non-survivors had higher PRISM-III and pSOFA scores and OI and PF ratios at 1–4 and 24 h (Table 1). Patients with indirect pARDS had a higher mortality rate than those with direct pARDS [57.4% vs. 37.1%, odds risk: 2.283 (1.222–4.2663), *p* = 0.009] (Table 1). Patients who were not malnourished exhibited better survival outcomes than undernourished children [61.1% vs. 45.8%, odds risk: 0.717 (0.520–0.988), *p* = 0.05].

**Table 1:** Comparison of baseline characteristics and ventilator parameters between survivors and non-survivors.

Variables median (IQR) *n* (%)	Survivors (*n* = 104)	Non-survivors (*n* = 81)	*p*-value
Age in years	1 (0.40–3.90)	1.60 (0.40–5.80)	0.295
Males	53 (51%)	55 (67.90%)	0.020
PRISM-III	8 (6.25–10)	9 (7–12.50)	0.005
pSOFA 0	7 (6–9)	9 (7–11)	<0.001
pSOFA 24	6 (5–8)	11 (8–13)	<0.001
Direct pARDS	78/124 (62.90%)	46/124 (37.10%)	0.009
Indirect pARDS	26/61 (42.60%)	35/61 (57.30%)	
Diagnosis			
Pneumonia	76 (73%)	46 (56.70%)	0.02
Sepsis with MODS	10 (9.60%)	15 (18.50%)	0.07
Severe dengue	15 (14.40%)	18 (22.20%)	0.17
Others	3 (2.80%)	2 (2.40%)	0.86
Malnutrition (%)	27/104 (26%)	32/81 (39.5%)	0.05
PaO_2_/FiO_2_ (1–4 h)	159.50 (126–189.75)	133 (104–172)	0.005
PaO_2_/FiO_2_ (24 h)	219.50 (161.25–242.75)	119.50 (83.05–197.50)	<0.001
FiO_2_ (1–4 h)	90 (75–95)	90 (82.50–100)	0.015
FiO_2_ (24 h)	67.50 (61.25–90)	95 (75–100)	<0.0001
OI (1–4 h)	8.10 (5.89–10.55)	8.80 (6.70–14.70)	0.028
OI (24 h)	5.05 (4.10–8.18)	11.70 (5.65–17.15)	<0.001
Tidal volume 1–4 h (ml/kg)	6 (6–7)	6.50 (6–7.50)	0.207
Tidal volume at 24 h (ml/kg)	6.45 (6–7.45)	6.40 (6–7.30)	0.896
PEEP 1–4 h (cmH_2_O)	6 (5–6)	6 (6–7)	0.022
PEEP 24 h (cmH_2_O)	6 (5–6)	6 (6–7)	0.001
PIP 1–4 h (cmH_2_O)	18 (16–22)	22 (18–24)	0.002
PIP 24 h (cmH_2_O)	16 (14–20.75)	23 (19.50–26.50)	<0.001
C_RS_ 1–4 h (ml/cmH_2_O/kg)	0.50 (0.38–0.66)	0.46 (0.36–0.58)	0.169
C_RS_ 24 h (ml/cmH_2_O/kg)	0.60 (0.43–0.82)	0.42 (0.30–0.55)	<0.0001
Respiratory rate/minute (1–4 h)	30 (28–35)	30 (30–40)	0.279
Respiratory rate/minute (24 h)	30 (26.50–40)	30 (30–40)	0.200

PaO_2_, partial pressure of oxygen; FiO_2_, fraction of inspired oxygen.

### MP, MP_BW_, MP_CRS_ and ME_BW_, and relationship with outcome measures

MP_BW_, MP_CRS_, and ME_BW_ but not MP were significantly higher among non-survivors at 1–4 h and after 24 h (Table 2). MP at 24 h was significantly higher in non-survivors only in mild pARDS (*p* < 0.022). MP_BW_, MP_CRS_, and ME_BW_ at 24 h were significantly higher in non-survivors with mild and moderate but not severe pARDS (Table 3).

**Table 2:** Comparison of mechanical power, normalized mechanical power, and dynamic driving pressure between survivors and non-survivors.

Variables median (IQR) *n* (%)	*N* = 185	Survivors (*n* = 104)	Non-survivors (*n* = 81)	*p*-value
Δ*P*_dyn_ 1–4 h (cmH_2_O)	14 (11 to 17)	12 (11 to 16)	15 (12 to 17)	0.008
Δ*P*_dyn_ 24 h (cmH_2_O)	13 (10 to 17)	11 (8 to 15)	16 (13 to 20)	<0.001
Change in Δ*P*_dyn_ over 24 h (cm H_2_O)	−1 (−3 to 2)	−2 (−4 to 0)	2 (−2 to 4)	<0.001
MP 1–4 h (J/min)	3.95 (2.32 to 6.43)	3.72 (2.12 to 5.82)	4.17 (2.62 to 7.96)	0.097
MP 24 h (J/min)	3.99 (2.12 to 6.89)	3.29 (1.91 to 5.37)	4.97 (2.66 to 8.23)	0.003
MP_BW_ 1–4 h (J/min/kg)	0.39 (0.31 to 0.49)	0.36 (0.30 to 0.48)	0.42 (0.33 to 0.57)	0.018
MP_BW_ 24 h (J/min/kg)	0.38 (0.29 to 0.53)	0.33 (0.27 to 0.46)	0.49 (0.37 to 0.57)	<0.001
Change in MP_BW_ over 24 h (J/min/kg)	−0.02 (−0.06 to 0.08)	−0.03 (−0.76 to 0.006)	0.06 (-0.03 to 0.13)	<0.001
ME_BW_ 1–4 h (mJ/kg)	12 (10 to 15)	12 (9 to 14)	13 (11 to 16)	0.006
ME_BW_ 24 h (mJ/kg)	12 (9 to 16)	11 (8 to 13)	14 (12 to 18)	<0.0001
Change in ME	0.70 (−1.60 to 1.60)	−1.20 (−1.80 to −0.10)	0.001 (−0.10 to 3.60)	<0.0001
MP_CRS_ at 1–4 h (J/min/ml/cmH_2_O)	1.09 (0.75 to 1.50)	0.96 (0.71 to 1.45)	1.22 (0.85 to 1.69)	0.015
MP_CRS_ at 24 h (J/min/ml/cmH_2_O)	1.03 (0.56 to 1.74)	0.73 (0.50 to 1.12)	1.63 (1.04 to 2.08)	<0.0001
Change in MP_CRS_ (J/min/ml/cmH_2_O)	0.11 (−0.44 to 0.32)	0.22 (0.01 to 0.37)	−0.38 (−0.61 to 0.12)	<0.0001

**Table 3:** Comparisons of MP, MP_BW_, MP_CRS_, and ME_BW_ between survivors and non-survivors in mild, moderate, and severe pARDS subgroups at 24 h.

Variables	Survivors	Non-survivors	*p*-value
Mild ARDS (*n*)	*n* = 52	*n* = 37	
MP (J/min)	4.13 (2.64–7.07)	6.17 (3.67–10.58)	0.022
MP_BW_ (J/kg/min)	0.45 (0.37–0.53)	0.54 (0.459–0.750)	0.008
MP_CRS_ (J/min/ml/cmH_2_O)	0.59 (0.48–0.87)	1.24 (0.947–1.699)	<0.001
ME_BW_ (mJ/kg/breath)	14.47 (12.00–16.97)	18.20 (15.31–23.43)	0.002
Moderate ARDS (*n*)	*n* = 46	*n* = 30	
MP (J/min)	4.70 (2.31–7.12)	6.07 (3.99–10.81)	0.150
MP_BW_ (J/kg/min)	0.44 (0.38–0.58)	0.63 (0.55–0.70)	<0.001
MP_CRS_ (J/min/ml/cmH_2_O)	0.85 (0.56–1.23)	1.74 (1.26–2.05)	<0.001
ME_BW_ (mJ/kg/breath)	15.28 (12.88–17.65)	18.45 (16.51–20.35)	<0.001
Severe ARDS (*n*)	*n* = 6	*n* = 14	
MP (J/min)	8.63 (5.78–11.29)	6.27 (3.10–9.72)	0.602
MP_BW_ (J/kg/min)	0.629 (0.55–0.87)	0.67 (0.58–0.84)	1.000
MP_CRS_ (J/min/ml/cmH_2_O)	1.44 (1.31–1.49)	2.23 (1.90–2.59)	0.274
ME_BW_ (mJ/kg/breath)	19 (18.16–22.26)	20.35 (18.50–25.14)	0.869

**Figure 2:**
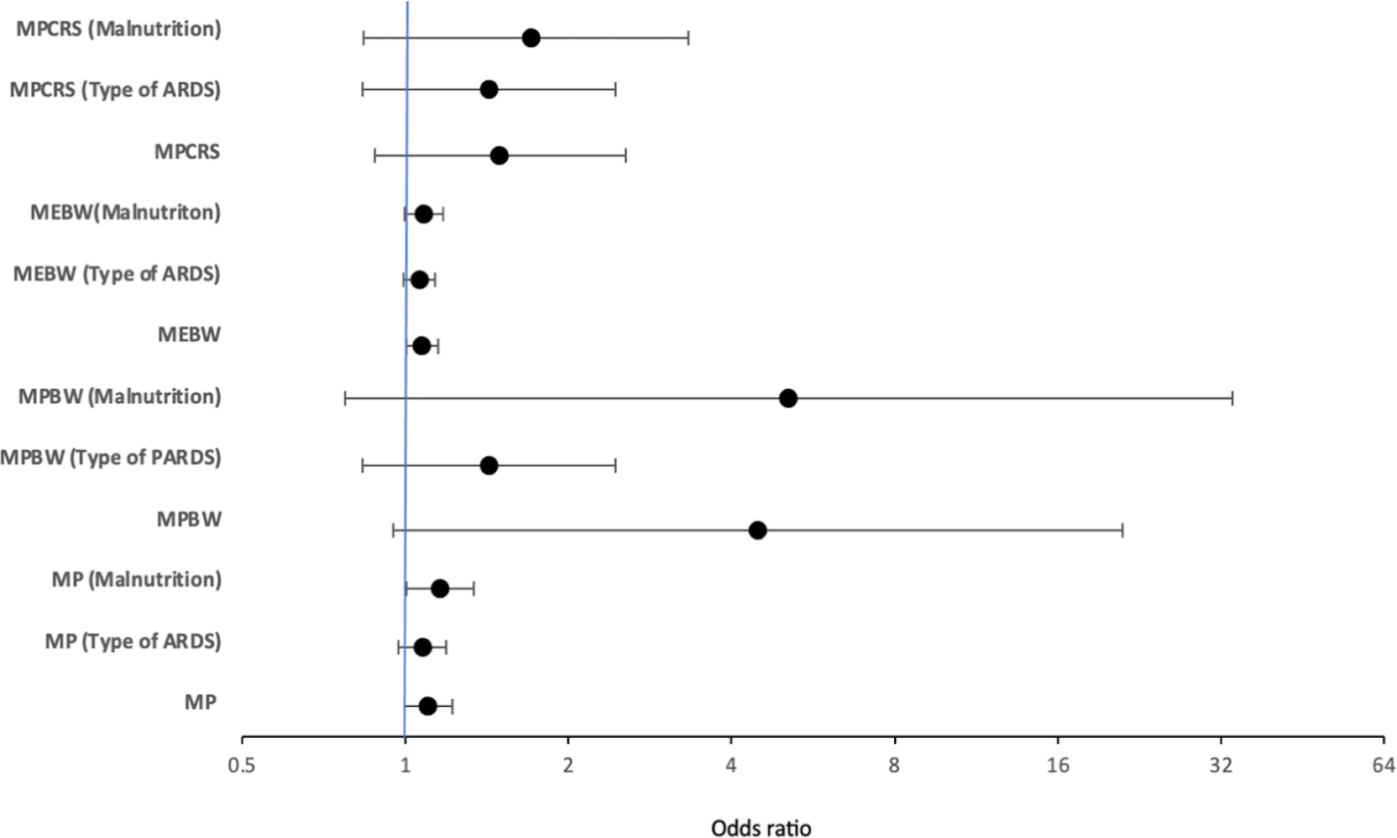
Figure depicting adjusted odds of multivariable logistic regression models of MP, MP_BW_, MP_CRS_, and ME_BW_ adjusted for age, organ failure score, oxygenation index, and gender, along with the change in adjusted odds after considering the type of pARDS and the presence of malnutrition at 24 h. MP, mechanical power; MP_BW_, MP normalized to body weight; MP_CRS_, MP normalized to respiratory compliance; ME_BW_, mechanical energy; ARDS, paediatric acute respiratory distress syndrome or pARDS.

**Figure 3:**
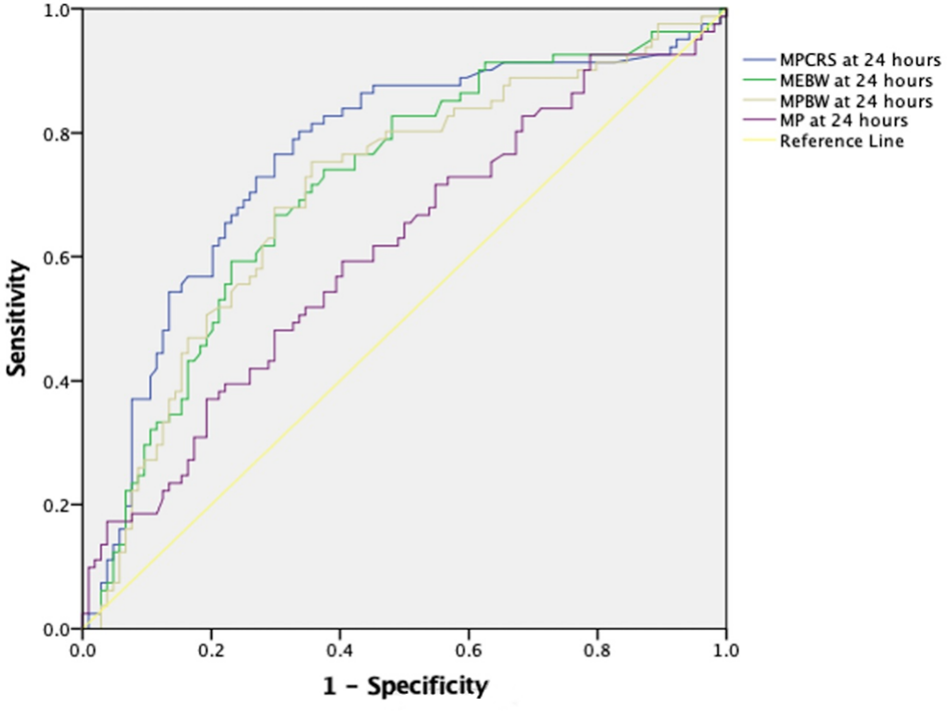
Receiver operating characteristic curve demonstrating MP, ME_BW_, MP_BW_, and MP_CRS_ at 24 h with 28-day mortality. The AUC was highest for MP_CRS_ (AUC: 0.759, 95% CI: 0.686–0.832), followed by ME_BW_ (AUC: 0.713, 95% CI: 0.637–0.788), MP_BW_ (AUC: 0.703, 95% CI: 0.626–0.780), and MP (AUC: 0.611, 95% Cl: 0.529–0.693). MP, mechanical power; MP_BW_, MP normalized to body weight; MP_CRS_, MP normalized to respiratory compliance; ME_BW_, mechanical energy.

Multivariable logistic regression analysis showed that only ME_BW_ but not MP, MP_BW_, or MP_CRS_ at 24 h was independently associated with mortality [adjusted odds ratio: 1.072 (1.002–1.147), *p* = 0.044] after adjusting for age, gender, pSOFA at 24 h, and OI at 24 h (Figure 2). When the model was adjusted for the type of pARDS, ME_BW_ was not independently associated with mortality [adjusted odds ratio: 1.061 (0.992–1.136), *p* = 0.085] (Figure 2), while indirect pARDS exhibited an increased risk of mortality [adjusted odds ratio: 2.424 (1.046–5.616), *p* = 0.039] (Supplementary Table S4a–d). When malnutrition was also included in addition to other parameters like age, gender, pSOFA, OI, and type of pARDS in the multivariable regression analysis, only MP at 24 h was independently associated with mortality (Supplementary Table S5a–d). ROC analysis of parameters at 24 h showed that the area under the curve (AUC) was the largest for MP_CRS_ (AUC: 0.759, 95% CI: 0.686–0.832), followed by ME_BW_ (AUC: 0.713, 95% CI: 0.637–0.788), MP_BW_ (AUC: 0.703, 95% CI: 0.626–0.780), and MP (AUC: 0.611, 95% CI: 0.529–0.693). The best cutoff value for MP_CRS_ was 1.019 (J/min/ml/cmH_2_O), with a sensitivity of 76.5% and a specificity of 69.2%. The cutoff value for ME_BW_ of 14 mJ/kg had the best sensitivity and specificity, although the sensitivity was only 75% and the specificity was 65%. Mortality rates among patients with ME_BW_ > 14 mJ/kg were significantly higher (52.6% vs. 21.2%, *p* < 0.001) (Figure 3).

**Figure 4:**
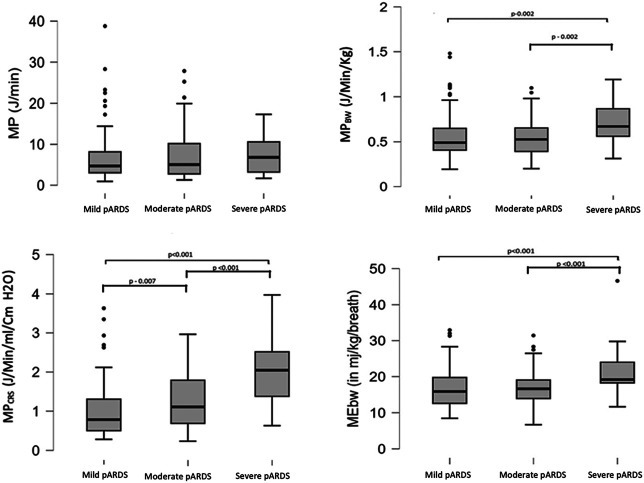
Comparison of MP, MP_BW_, MP_CRS_, and ME_BW_ with mild, moderate, and severe pARDS. MP, mechanical power; MP_BW_, MP normalized to body weight; MP_CRS_, MP normalized to respiratory compliance; ME_BW_, mechanical energy; pARDS, pediatric acute respiratory distress syndrome.

There was a linear relationship between pARDS severity and MP_BW_, MP_CRS_, and ME_BW_ but not for MP (Figure 4). All variables significantly correlated with OI, with MP_CRS_ having the highest, albeit modest, correlation coefficient (Supplementary Table S3).

Only MP_CRS_ at 1–4 and 24 h but not MP, MP_BW_, or ME_BW_ was significantly but modestly correlated with VFD-28 (Supplementary Table S6).

## Discussion

To our knowledge, this is the first study that has examined the relationship between normalization of mechanical power by body weight and respiratory compliance with pARDS severity strata and outcomes. We found a clear relationship between pARDS severity strata and power normalized to body weight and compliance but not for non-normalized power. However, we could not demonstrate an independent association between normalized mechanical power and mortality on multivariable analyses, while non-normalized MP at 24 h was associated with mortality. This association was influenced by the prevalence of malnutrition in our cohort.

The potential advantage of using MP lies in its ability to provide individualized quantification of “pressure” and “non-pressure” variables (Vt, RR, Δ*P*, PEEP, I:E ratio, flow) (1, 2, 10). In adults, high MP has been associated with increased intensive care unit mortality, especially when MP was normalized to C_RS_ (5, 15). Our observations agree with previous secondary analyses of the PARDIE study data (11). In that particular study, MP was normalized to predicted body weight and was only independently associated with mortality when children who died due to neurologic reasons were excluded. Interestingly, in our study, MP was not associated with mortality when malnutrition was not considered. Only ME_BW_ was associated with mortality; that association was also lost when adjusted for the type of pARDS (direct or indirect). When malnutrition was also included in addition to other parameters like age, gender, pSOFA, oxygenation index, and type of pARDS in the multivariable regression analysis, only MP at 24 h was independently associated with mortality. This means that malnutrition is an essential factor that needs to be considered while assessing the impact of MP in pARDS. Children with average or low body mass index have been shown to have lung volumes reflecting measured or actual body weight (21). In one study, Vt measured in ml/kg was found to be higher when using ideal body weight than actual body weight, particularly for overweight and obese children (22). Due to the prevalence of malnutrition, we used actual body weight to calculate Vt to be delivered during ventilation and to calculate ME_BW_, MP, and its derivatives in our cohort. Like our study, Kneyber et al. calculated mechanical energy using actual body weight as they had no obesity in their cohort (12).

In their study, Bhalla et al. reported that MP_BW_ was independently associated with VFD-28 only in children <2 years of age (11). However, in our study, only MP_CRS_ at 1–4 and 24 h but not MP, MP_BW_, or ME_BW_ was significantly correlated with VFD-28.

In our study population, we observed low levels of PEEP being used, and this may have affected the findings of our study, as inherently lower levels of PEEP lead to a higher dynamic airway pressure gradient and, thus, higher lung stress and can be associated with increased mortality (11, 23). However, the use of lower levels of PEEP is described in other studies in the pediatric age group as well (12, 24, 25). Due to the nature of our study, we have no data on plateau pressure and cannot estimate actual lung stress in our cohort. The dynamic airway pressure gradient was significantly higher among non-survivors, exceeding potentially injurious thresholds and confounding the present study's findings.

The respiratory rate changes with age in children, which may challenge the interpretation of MP (12). For this reason, it was proposed previously to calculate energy normalized to body weight per breath (12). However, RR is an essential component of the equation of MP and a cause for significant VILI (4–6). The tradeoffs of removal of RR from the MP equation for calculation of ME_BW_ are not known, especially since RR is a strong predictor for outcome in adults with ARDS (5). Some authors have, therefore, normalized MP to lung size or the degree of well-inflated tissue and lung compliance and showed that these normalized values were independently associated with mortality (15, 26). Together with our observations, this suggests that MP needs normalization or indexing considering factors such as age, weight, or lung volume (15, 27).

No single component of MP has been shown to significantly contribute to the overall MP (1). Such observations were also made in the secondary analyses of the PARDIE study data (11). This means there is no straightforward approach to reducing MP and improving patient outcomes. One group of investigators reported improved outcomes in mild-to-moderate ARDS when Vt was increased with a concomitant reduction in RR to maintain a safe minute ventilation (28). Experimental work has shown that Vt should be kept at safe lower levels, even low MP, to protect the lungs from VILI (6). Since our study is observational, we cannot conclude that lowering MP would lead to improved outcomes in pARDS. This warrants further study.

Our study has some limitations that need to be discussed. First, being a single-center study, it limits generalizability. Second, our study's mortality rate of pARDS is higher than published studies in the West (29). Recent meta-analyses report that mortality in pARDS differs depending on geographical location, with Asian countries being higher than the West due to various factors like associated sepsis, immunodeficiency, malignancy, and socioeconomic and cultural background (29, 30). The reported mortality rates from Asian countries are similar to those reported in our study (29, 30). We also found malnutrition to be significantly associated with mortality in our study. Third, due to the study design, we could not evaluate the impact of the size of the endotracheal tube and airway resistance on the delivery of pressures and energy on the lung parenchyma. In a bench-side study, Ilia et al. showed that in a situation of no zero-flow (decelerating flow) in the presence of increasing airway resistance, the pressure drop between PIP and alveolar pressures increases (31). Thus, higher airway resistance associated with a smaller endotracheal tube or bronchospasm may not allow alveolar pressures to reach PIP. Fourth, we could not assess the influence of chest wall elastance, as transpulmonary pressures were not measured. Poor chest wall compliance may significantly impact the measurements (32). However, a recent study has suggested that the transpulmonary driving pressure was an equivalent predictor of outcomes to the driving pressure measured using airway pressures (33). Fifth, dynamic driving pressure (Δ*P*_dyn_) was calculated as the difference between PIP and PEEP. Differences between PIP and PEEP in PC mode can significantly overestimate the actual (transpulmonary) driving pressure if the inspiratory flow does not reach a zero-flow state before the end of inspiration (25). However, earlier studies have used the difference between PIP and PEEP to calculate dynamic driving pressure, similar to our study (11, 24, 34). Moreover, Becher's equation, used in our study, is validated to calculate MP in PC mode without applying inspiratory hold (13, 20, 35, 36). Finally, although patients in our cohort were well sedated, and if needed, even paralyzed, in the first 24–48 h of identification of pARDS, there is still a possibility of some patients having some spontaneous breathing during the study period. In PC mode, the Vt may vary if there is any spontaneous breathing, thus potentially affecting the accurate measurement of MP and ME_BW_.

## Conclusion

Normalization of MP is better related to outcomes and severity of pARDS than non-normalized MP. Malnutrition can be a significant confounding factor in resource-limited settings.

## Data Availability

The original contributions presented in the study are included in the article/Supplementary Material, further inquiries can be directed to the corresponding author.
