# Supplementary material for: Mechanical power and normalized mechanical power in pediatric acute respiratory distress syndrome
Source: Front Pediatr. 2024 Jan 16;12:1293639. doi: 10.3389/fped.2024.1293639 (PMC10829106; doi:10.3389/fped.2024.1293639)
Supplement: Supplementary file 1 [file Table1.docx]

Table of Contents

[**1.** **Supplemental Digital Content - 1** 2](#_Toc139149234)

[**2.** **Supplementary Digital Content-2** 10](#_Toc139149235)

[**3.** **eTable-1:** Distribution of baseline characteristics and outcome variables. 11](#_Toc139149236)

[**4.** **eTable 2. Distribution of baseline characteristics and outcome variables between survivors and non-survivors in mild, moderate, and severe pARDS** 13](#_Toc139149237)

[**5.** **e Table 3: Correlation of mechanical power and normalized mechanical power indices with oxygenation index at 24-hours** 14](#_Toc139149239)

[**6.** **e-Table.4a. Multivariable logistic regression modelling analysis to determine the association MP_CRS_ adjusted for age, sex, oxygenation index, organ dysfunction score and type of ARDS** 15](#_Toc139149311)

**7.** [**e-Table.4b Multivariable logistic regression modelling analysis to determine the association MP_BW_ adjusted for age, sex, oxygenation index, organ dysfunction score and type of ARDS** 16](#_Toc139149312)

**8.** [**e-Table.4c - Multivariable logistic regression modelling analysis to determine the association MP adjusted for age, sex, oxygenation index, organ dysfunction score and type of ARDS** 17](#_Toc139149313)

**9.** [**e-Table.4d Multivariable logistic regression modelling analysis to determine the association ME_BW_ adjusted for age, sex, oxygenation index, organ dysfunction score and type of ARDS** 18](#_Toc139149314)

**10.** e-Table 5a Multivariable logistic regression modelling analysis to determine the association ME_BW_  adjusted for age, sex, oxygenation index, organ dysfunction score, type of ARDS and MALNUTRITION. …………………………………………………………………………………………………………..19

**11.** e-Table5b **Multivariable logistic regression modelling analysis to determine the association MP at 24 hrs adjusted for age, sex, oxygenation index, organ dysfunction score, type of ARDS and MALNUTRITION………………………………………………………………… 19**

**12. e-Table 5c Multivariable logistic regression modelling analysis to determine the association MP_BW_ at 24 hrs adjusted for age, sex, oxygenation index, organ dysfunction score, type of ARDS and MALNUTRITION…………………………………………………………………………………………………… 20**

**13. e-Table 5d Multivariable logistic regression modelling analysis to determine the association MP_CRS_ at 24 hrs adjusted for age, sex, oxygenation index, organ dysfunction score, type of ARDS and MALNUTRITION…………………………………………………………… 20**

**14. e Table 6: Correlations of 28-day ventilator-free days with mechanical power, normalized mechanical power……………………………………………………………………………. 21**

**15. e-Figure1: showing probability of mortality at day-28 with mechanical energy at 24 hours …………………………………………………………………………………………………………………….. 23**

**Supplemental Digital Content – 1**

MECHANICAL POWER AND NORMALIZED MECHANICAL POWER IN PEDIATRIC ACUTE RESPIRATORY DISTRESS SYNDROME

**Definitions:**

**28-day ventilator-free days:** Number of days of unassisted breathing till day 28 of recruitment, assuming a child survives for at least two consecutive days after initiation of unassisted breathing and remains free of assisted breathing.T

**28-day mortality:** Mortality within 28 days

Within 28 days of admission, or 28 days after diagnosis of pARDS, or 28 days of mechanical ventilation

The weight of the patients was measured directly at the time of admission, and tidal volume was indexed based on the measured weight at admission.

**PALICC definition of pARDS:^i^**

1. Children above 30 days age up to 18 years after excluding patients with perinatal lung disease.
2. Duration of illness less than 7 days from known clinical insult.
3. The respiratory failure not fully explained by cardiac failure or fluid overload.
4. Chest imaging finding of new infiltrate(s) consistent with acute lung parenchymal disease
5. In non-invasively ventilated patient (on face mask or BiPAP) with
6. CPAP> 5cmH2O PaO2/FiO_2_ < 300 or SF ratio < 264
7. In invasively ventilated patients:
   1. Mild category: 4 < Oxygen Index (OI) < 8. OR. 5 < Oxygen saturation Index (OSI) < 7.5
   2. Moderate category: 8 < OI < 16 OR 7.5 < OSI < 12.3
   3. Severe category: OI > 16. OR OSI > 12.3
8. In children with Cyanotic Heart Disease and/or left ventricular dysfunction: Standard criteria as mentioned above for age, timing, origin of lung edema and chest imaging with an acute deterioration in oxygenation not explainable by underlying cardiac disease or left ventricular dysfunction.
9. In children with chronic lung disease: Standard criteria as mentioned above for age, timing, origin of lung edema and chest imaging consistent with new infiltrates and acute deterioration in oxygenation from baseline which meet oxygenation criteria mentioned above.

**Exclusion criteria:**

1. Children with pARDS ventilated for less than 24 hours
2. High-Frequency Oscillatory ventilation (HFOV) or Extra Corporeal Membrane Oxygenation (ECMO) within 24 hours of diagnosis of pARDS
3. Non-invasive ventilation
4. Incomplete medical records
5. Neuromuscular diseases
6. Malignancy
7. Primary immunodeficiency

Children with these diseases (Neuromuscular diseases, malignancies, primary immunodeficiencies) were excluded because they can independently affect the out- come parameters like duration of ventilation due their underlying clinical condition.

**Data Collected.**

Because this was a retrospective study there was no daily screening done. From the data collected, the investigators verified whether each patient met the criteria for pARDS based on the PALICC guidelines.

The ventilator parameters and patient’s clinical parameters are noted by the bedside nurses in the patient medical records during the ICU stay. The medical records are stored as hard copies as well as scanned soft copies in the hospital medical records department. The investigators extracted the data required from these medical records. The accuracy of the data extracted from the medical records were verified by 2 of the investigators (Dr. Pallavi and Dr. Vishnuvardhan, under supervision of Dr Farhan Shaikh).

From the review of ventilation charts, 2 of the investigators determined whether the data fit the criteria for the diagnosis of PARDS as per the PALICC guidelines. This was done both within first 1-4 hours of intubation and then at 24 hrs if the patient was still on conventional mode of ventilation. If they met all the inclusion criteria, they were enrolled in the study.

Clinical and mechanical ventilator parameters were retrospectively collected from the ventilator monitoring charts, firstly, 0-4 hours of diagnosis of pARDS (“0-4 hours” parameters) and subsequently 24-hours later (“24-hours” parameter). Clinical parameters collected were Pediatric Risk of Mortality (PRISM-III) score at the end of 24-hours, Vasotropic Inotrope Score (VIS), Sequential Organ Failure Assessment (pSOFA) score ratio of PaO2 to FiO_2_ (PF-Ratio), Oxygen Index (OI). Mechanical ventilator’s parameters collected were FiO_2_, Peak inspiratory pressure (PIP), Peak End expiratory Pressure (PEEP), Respiratory rate (RR), Inspiratory time (Ti), Ratio of inspiratory and expiratory times (IE-ratio), Tidal Volume (Vt), Dynamic compliance (Cdyn). Airway resistance (Raw) was not calculated as it was not registered in the ventilator charts.

MP was calculated by the surrogate formula suggested by Becher et al ^iv^

MP = 0.098 x RR x V_T_ x (ΔP + PEEP)

ΔP in this equation suggested by Becher is difference between PIP and PEEP. We defined ΔP as “dynamic driving pressure (ΔP_dyn_) as described by Abdul Rauf et al^vi^. Normalized MP (NorMP) was calculated by normalizing MP to body weight. MP Normalized to respiratory system compliance was calculated by dividing MP by the Respiratory system compliance. Respiratory system Complinace was Vt/ ΔP_dyn_,.

Mechanical energy was calculated by removing RR and normalizing Vt to body weight from Becher’s equation as proposed by Kneyber et al.^vii^

**Ventilatory Strategy:**

The mechanical ventilators used were GE CARESCAPE R860. Software version 10 (infant Pediatric and adult), Manufacturer: Datex Ohmeda Inc. Made in USA. And MAQUET (GETINGE Group) SERVO-i v8.0 (infant and Pediatric) (Manufacturer. Maquet critical care AB. Sweden). In both GE CARESCAPE R860 and MAQUET Servo-I and Servo-S ventilators, circuit compliance, resistance and leak are measured and compensated each time at the initiation of the ventilation. In both the ventilators the inspiratory tidal volume (VTi) is measured by the inspiratory sensor and displayed on the screen of the ventilators. In both the ventilators the inspiratory flow sensors are automatically zeroed each time when the ventilator is started. In our unit, all the ventilators undergo preventive maintenance and calibrations once every year as per the manufacturer's recommendations.

All children were ventilated in Pressure Control (PC) mode. The unit policy is to ventilate children at Vt of 6-8ml/kg of mild to moderate pARDS and 3-6 ml/kg if moderate to severe pARDS with an aim to keep PIP below 28 cmH_2_O and if there is evidence of poor chest wall compliance then to try keeping PIP below 30-32 cmH_2_O. The initial Vt selected was 6ml/kg for all children, and while doing so, resulting PIP was monitored closely. In situations where the PIP was reaching above 28 cmH2O, the Vt was reduced gradually up to even 3ml/kg so that the PIP comes below the safe limit of 28 cmH_2_O. Both inspiratory and expiratory Vt was measured by the ventilator. The nurses write down the “expiratory Vt” in their ventilator chart. We have taken “expiratory Vt” for our calculations from the ventilator chart. Respiratory rates were selected appropriate for the age and clinical condition and was titrated by monitoring the Flow-Time scalar on the ventilator screening. Inspiratory time was adjusted to allow inspiratory flow to touch the baseline before the expiratory flow started. Ti was shorter than the expiratory time (Te). PEEP was first selected at 5 to 6 cmH_2_O and then titrated watching the Pressure-Volume loop ensuring a PEEP achieving best SpO_2_ at least hemodynamic disturbance, and best dynamic compliance with the least PIP. Recruitment Maneuver was not employed on a routine basis. The recruitment maneuver was used only in children with severe pARDS (OI > 16) , where there was clinical “potential for recruitment” (Lungs of child with severe pARDS were considered “having potential for recruitment” if increasing the PEEP by 2-4 cmH_2_O was showing improvement in oxygen saturation, improvement in lung compliance without increasing Pplat without any hemodynamic compromise). If PIP was going higher than 28-32 cm H_2_O, then targeted Vt was reduced to 3-4ml/kg to keep it below 28-30cmH2O. Ppeak/PIP of 30-32 was tolerated in those with increased chest wall elastance (e.g. chest wall edema, massive abdominal distension, obesity etc.). Acceptable SpO2 was 88-92%%. Permissive hypercapnia was allowed where necessary. We used Morphine infusion for sedation of our ventilated children. In the first 24-48 hours after intubation the targeted sedation was of Ramsay score 3 to 4. To achieve better sedation, intermittent boluses of IV Midazolam and / or Ketamine was also administered to stop spontaneous breathing and patient-ventilator asynchrony during the first 24-48 hours of “acute” phase of mechanical ventilation. IV Pancuronium was administered if any asynchrony was noticed in the first 24 hours of mechanical ventilation. Thus, all children were well sedated and even paralyzed if needed, to minimize patient ventilator asynchrony and spontaneous breaths during the first 24-48 hours period of data collection for calculation for ventilator parameters in first 3 to 4 hours of inclusion in the study and subsequently after 24 hours.

| **Adjunctive Therapies** | |
| --- | --- |
| **Fluid Overload** | If Fluid Overload (F.O.) > 10%, Lasix infusion and fluid restriction was used. If FO persisting or worsening, then depending on clinical situation RRT (PD/SLED or CRRT) |
| **Antibiotic therapy** | As per unit policy based on antibiotic stewardship program of the unit |
| **Steroids** | Steroids are not used in our unit for treatment of pARDS per se. However steroids may be administered as part of management of associated clinical conditions like shock, HLH, other inflammatory conditions needing steroids |
| **Sedation Policy** | Sedation by Morphine infusion targeting Ramsay score of 3-4 and minimal spontaneous breathing and patient-ventilator asynchrony in the first 24-48 hours of “acute phase”. Top up sedation by bolus of Ketamine or Midazolam. |
| **NM Blockers** | NM Blockers may be used in severe pARDS in the first 24 hours of acute care to avoid asynchrony related to spontaneous breathing. |
| **Vasopressors and inotropes** | As per unit policy based on clinicians discretion |
| **Enteral nutrition** | As per unit’s policy. Early enteral nutrition is encouraged wherever clinically possible. |
| **Physiotherapy** | Not utilized in initial acute phase of pARDS. May be utilized in select cases. |
| **Bronchodilators** | Not utilized routinely. May be used in select cases depending upon the need |
| **ETT** | Cuffed ETT shall be used. |
| **Cardiac evaluation** | Regular evaluation of cardiac evaluation by bed side Echo shall be performed to assess cardiac functions (chamber size, contractility) and status of the inferior vena cava. |
| **Weaning and extubation** | Patient shall be shifted to weaning mode (SIMV-VC or PC mode) and when the FiO2 is 0.4 and child is awake and cooperative breathing spontaneously, a Spontaneous Breathing Trial shall be used for assessment of readiness of extubation. Those fit for extubation shall be extubated to either Oxygen by nasal canula, or face mask of HFNC or CPAP depending upon the clinical condition |

References:

Khemani RG, Smith LS, Zimmerman JJ, Erickson S; Pediatric Acute Lung Injury Consensus Conference Group. *Pediatr Crit Care Med. 2015 Jun;16(5 Suppl 1):S23-40.*

ii. . Spicer, A. C. et al. A simple and robust bedside model for mortality risk in pediatric patients with acute respiratory distress syndrome. *Pediatr. Crit. Care Med.* **17**, 907–916 (2016).

iii. Gattinoni L, Tonetti T, Cressoni M, Cadringher P, Herrmann P, Moerer O, Protti A, Gotti M, Chiurazzi C, Carlesso E, Chiumello D, Quintel M. Ventilator-related causes of lung injury: the mechanical power. *Intensive Care Med. 2016 Oct;42(10):1567-1575.*

*iv.* Becher T, van der Staay M, Schädler D, et al: Calculation of mechanical power for pressure-controlled ventilation. *Intensive Care Med.* 2019 Sep;45(9):1321-1323.17

v. Amato MB, Meade MO, Slutsky AS, et al: Driving pressure and survival in the acute respiratory distress syndrome. *N Engl J Med.* 2015 Feb 19;372(8):747-55.

*vi ^.^ Abdul Rauf, Anil Sachdev, Shekhar T Venkataraman and Veronique Dinand. Dynamic Airway Driving Pressure and Outcomes in Children With Acute Hypoxemic Respiratory Failure. Respiratory Care March 2021,  66 (3) 403-409; DOI:* [*https://doi.org/10.4187/respcare.08024*](https://doi.org/10.4187/respcare.08024)

vii. Kneyber MCJ, Ilia S, Koopman AA, et al: Energy transmission in mechanically ventilated children: a translational study. *Crit Care.* 2020 Oct 7;24(1):601.

## **Supplementary Digital Content-2**

## **eTable-1:** Distribution of baseline characteristics and outcome variables.

| Variables  Median (IQR)  n (%) | All (n-185) | Mild ARDS  (n – 89) | Moderate ARDS  (n – 76) | Severe ARDS  (n – 20) | p-value |
| --- | --- | --- | --- | --- | --- |
| Age in years | 1.3 (0.4 – 5.0) | 1 (0.41 – 5.0) | 1.54 (0.43 – 5.49) | 1.66 (0.27 – 3.75) | 0.680 |
| PRISM III | 8 (7 – 12) | 8 (6.0 – 9.0) | 9 (7.0 – 12.0) | 9.5 (8.0 – 12.0) | 0.004 |
| pSOFA at 1-4 hrs | 8 (6 – 10) | 7 (5.0 – 9.0) | 8.5 (7.0 – 10.0) | 9.5 (8.0 – 10.75) | <0.0001 |
| pSOFA 24 | 8 (5 – 12) | 6 (5.0 -10.0) | 8.0 (6.0 – 12.0) | 11 (7.25 – 13.75) | <0.0001 |
| PaO2/FiO2 (1 – 4 hours) | 149 (116 – 185) | 177.5 (152.2 – 214.5) | 127.0 (110.0 – 149.75) | 91 (62.77 – 110) | <0.0001 |
| PaO2/ FiO2 (24 hours) | 180 (105 – 232) | 224.0(159.75 – 254.25) | 154.5 (94.0 – 216.75) | 91 (57.62 – 159.0) | <0.0001 |
| OI (1 – 4 hours) | 8.3 (6.3 – 12.3) | 5.95 (5.20 – 6.9) | 10.8 (8.9 – 12.7) | 19.15 (16.9 – 26.45) | <0.0001 |
| OI (24 hours) | 6.1 (4.3 – 12.6) | 4.6 (3.5 – 6.62) | 8.35 (5.12 – 14.52) | 19.55 (12.35 – 32.0) | <0.0001 |
| Tidal volume (1 -4 hours) (ml/Kg) | 6.2 (6, 7.14) | 6.3 (6.0, 8.0) | 6.0 (6.0, 7.0) | 6.45 (6.0, 7.1) | 0.028 |
| Tidal volume (24 hours) (ml/Kg) | 6.4 (6.0, 7.3) | 6.6 (6.0, 8.0) | 6.0 (6.0, 7.0) | 6.6 (6.0, 7.6) | 0.015 |
| Respiratory rates (1 - 4 hours) (breaths/minute) | 30 (30, 35) | 30 (30, 40) | 30 (28, 35) | 32.5 (30, 35) | 0.520 |
| Respiratory rates (24 hours) (breaths/minute) | 30 (30, 40) | 30 (30, 40) | 30 (28, 40) | 32.5 (30, 37.5) | 0.593 |
| PIP (cm H_2_O)  (1- 4 hours) | 20 (17, 23) | 18 (16, 21) | 22 (18, 24) | 24 (21, 27) | <0.0001 |
| PIP (Cm H_2_O) (24 hours) | 20 (15, 24) | 17 (14, 22) | 20 (16, 25) | 26 (22, 28.5) | <0.0001 |
| PEEP (Cm H_2_O)  1 - 4 hours | 6 (6, 7) | 6 (5, 6) | 6 (6, 7) | 6 (6, 8) | 0.004 |
| PEEP (Cm H_2_O)  24 hours | 6 (5, 7) | 6 (5, 7) | 6 (6, 7) | 6 (6,7) | <0.011 |
| ΔP_dyn_ (cm H_2_O)  1 - 4 hours | 14 (11, 17) | 12 (10, 15) | 15 (12, 17.7) | 17 (13.75, 20) | <0.0001 |
| ΔP_dyn_) (cm H_2_O) 24hours | 13 (10, 17) | 11 (9, 15) | 14 (11, 18) | 20 (15.5, 21.5) | <0.0001 |
| Cdyn (1 - 4 hour)  ml/kg/cm H_2_O | 0.5 (0.37, 0.60) | 0.55 (0.43, 0.72) | 0.44 (0.33, 0.54) | 0.39 (0.33, 0.49) | <0.0001 |
| Cdyn (24 hours)  ml/kg/cm H_2_O | 0.51 (0.38, 0.75) | 0.57 (0.43, 0.79) | 0.47 (0.35, 0.67) | 0.37 (0.30, 0.44) | <0.0001 |
| FiO2 (1-4 hours) (%) | 90 (80, 95) | 80 (70, 90) | 95 (90, 95) | 100 (95, 100) | <0.0001 |
| FiO2 (24 hours) (%) | 80 (65, 100) | 65 (60, 90) | 90 (70, 100) | 100 (87.5, 100) | <0.0001 |
| Required HFOV | 61 (32.9%) | 15 (16.3%) | 34 (44.73%) | 12 (60%) | <0.0001 |
| Required ECMO | 2 (1%) | 0 (0%) | 1 (1.31%) | 1 (5%) | - |
| Need for CRRT | 30 (16.2%) | 15 (16.85%) | 12 (15.7%) | 3 (15%) | 0.971 |
| Length of PICU stay | 7.5 (5.0 -10.0) | 7 (5 – 10) | 8 (6 – 11) | 8 (3 – 10.75) | 0.635 |
| 28-day Ventilator free days | 13.0 (0.0 – 22.7) | 18 (0 – 23) | 16.5 (0 – 20.75) | 0 (0 – 20.75) | 0.124 |
| 28-day mortality (%) | 81/185 (43.8%) | 37/89 (41.6%) | 30/76 (39.5%) | 14/20 (70%) | 0.045 |

*Foot note: IQR – interquartile range; PRISM III – Pediatric Risk of Mortality at 24hrs; pSOFA – pediatric Sequential organ failure assessment; PICU – Pediatric intensive care unit, OI – oxygenation Index ; PIP - Peak Inspiratory pressure, PEEP - Positive End Expiratory Pressure;* ΔP_dyn_ *- Dynamic Driving pressure;*  C_dyn_ *- Dynamic compliance; HFOV – High Frequency Oscillatory Ventilation; ECMO – Extracorporeal membrane oxygenation; CRRT – Continuous renal replacement therapy*

## **eTable 2. Distribution of baseline characteristics and outcome variables between survivors and non-survivors in mild, moderate, and severe pARDS**

| Variables  *Median (IQR)*  *n (%)* | Mild pARDS (n – 89) | | | Moderate pARDS (n – 76) | | | Severe pARDS (n – 20) | | |
| --- | --- | --- | --- | --- | --- | --- | --- | --- | --- |
|  | All | Survivors  (n – 52) | Non-survivors  (n – 37) | All | Survivors  (n – 46) | Non-survivors (n – 30) | All | Survivors  (n – 6) | Non-survivors (n – 14) |
| Age in years | 1 (0.41 – 5.0) | 0.91 (0.41 – 3.0) | 1.91 (0.37 – 6.2) | 1.54 (0.43 – 5.49) | 1.54 (0.39 – 5.68) | 1.53 (0.56 – 5.91) | 1.66 (0.27 – 3.75) | 1.7 (0.62 – 5.5) | 1.66 (0.25 – 3.75) |
| PRISM III | 8 (6.0 – 9.0) | 7 (5 – 8) | 9 (7 – 13) | 9 (7.0 – 12.0) | 9 (7 – 12) | 9.5 (7.75 – 13.25) | 9.5 (8.0 – 12.0) | 9.5 (7.75 – 13.5) | 9.5 (7.75 – 12.0) |
| pSOFA at 0-4 hrs | 7 (5.0 – 9.0) | 7 (5 – 8) | 9 (6 – 11) | 8.5 (7.0 – 10.0) | 8 (7 – 10) | 9(7.75 – 10) | 9.5 (8.0 – 10.75) | 8 (5.5 – 10.5) | 10 (8 – 11.25) |
| pSOFA 24 | 6 (5.0 -10.0) | 5 (4 – 6) | 10 (7 – 12) | 8.0 (6.0 – 12.0) | 7 (5 – 10) | 11 (8 – 13.25) | 11 (7.25 – 13.75) | 7 (6 – 10.25) | 12 (10 – 14) |
| PaO2/FiO2 (0 – 4 hours) | 177.5 (152.2 – 214.5) | 179 (159 – 206) | 173 (141 – 219) | 127.0 (110.0 – 149.75) | 133.5 (120.5 – 163.75) | 115 (93.75 – 130.75) | 91 (62.77 – 110) | 85 (57.15 – 124.75) | 91 (63.37 – 114.75) |
| PaO2/ FiO2 (24 hours) | 224.0(159.75 – 254.25) | 231 (186 – 255) | 188 (102.75 – 258.5) | 154.5 (94.0 – 216.75) | 198.5 (152.25 – 232.0) | 95 (72.75 – 125.25) | 91 (57.62 – 159.0) | 120.5 (64.25 – 175.75) | 86.5 (56.12 – 134.5) |
| OI (0 – 4 hours) | 5.95 (5.20 – 6.9) | 5.89 (5.2 – 6.5) | 6.46 (5.09 – 7.36) | 10.8 (8.9 – 12.7) | 9.85 (8.87 – 12.32) | 12.15 (9.4 – 14.3) | 19.15 (16.9 – 26.45) | 18.1 (16.62 – 20.0) | 22(16.9 – 29.4) |
| OI (24 hours) | 4.6 (3.5 – 6.62) | 4.3 (3.5 – 5.2) | 5.9 (3.85 – 12.0) | 8.35 (5.12 – 14.52) | 6.15 (4.8 – 8.75) | 13.7 (8.17 – 18.08) | 19.55 (12.35 – 32.0) | 13.6 (12.27 – 22.52) | 25.3 (12.8 – 32.35) |
| Required HFOV | 15 (16.3%) | 3 (20%) | 12 (80%) | 34 (44.73%) | 13 (38.23%) | 21 (61.76%) | 12 (60%) | 3 (25%) | 9 (75%) |
| Required ECMO | 0 (0%) | 0 (0%) | 0 (0%) | 1 (1.31%) | 1 (100%) | 0 (0%) | 1 (5%) | 1 (100%) | 0 (0%) |
| Need for CRRT | 15 (16.85%) | 2 (13.33%) | 13 | 12 | 6 | 6 | 3 | 1 | 2 |
| Length of PICU stay | 7 (5 – 10) | 8 (5.0 – 10.0) | 7 (5.0 – 9.5) | 8 (6 – 11) | 8 (6.0 – 11.0) | 6.5 (4.0 – 10.25) | 8 (3 – 10.75) | 10 (6.5 – 13.25) | 6 (2.75 – 10.5) |
| Length of hospital stay | 8.5 (6 – 12.75) | 11.0 (7.0 – 14.0) | 7 (5.0 – 9.5) | 9 (6 – 12.75) | 10.0 (8.0 – 14.25) | 6.5 (4.0 – 10.25) | 8.5 (3.0 – 14.0) | 13.5 (8 – 19.5) | 6 (2.75 – 12.5) |
| 28-day Ventilator free days | 18 (0 – 23) | 23 (20 – 25) | 0 (0 – 0) | 16.5 (0 – 20.75) | 21.0 (18.0 – 23.0) | 0 (0 – 0) | 0 (0 – 20.75) | 20.5 (12.75 – 21.5) | 0 (0 – 0) |

*IQR – interquartile range; PRISM III – Pediatric Risk of Mortality at 24hrs; pSOFA – Sequential organ failure assessment; OI – Oxygenation Index; PICU – Pediatric intensive care unit; HFOV – High Frequency Oscillatory Ventilation; ECMO – Extra Corporeal Membrane Oxygenation; CRRT- Continuous Renal Replacement Therapy*

## **e Table 3: Correlation of mechanical power and normalized mechanical power indices with oxygenation index at 24-hours**

| Correlation of Oxygenation index with | Spearman’s correlation coefficient (rho) | p-value |
| --- | --- | --- |
| Mechanical power (MP) | 0.254 | <0.0001 |
| Mechanical power normalized to body weight (MP_BW_) | 0.365 | <0.0001 |
| Mechanical Energy (ME_BW_) | 0.363 | <0.0001 |
| Mechanical power normalized to respiratory system compliance (MP_CRS_) | 0.598 | <0.0001 |

MP – mechanical power, MP_BW_ – Mechanical power normalized to body weight, MP_CRS_ – Mechanical power normalized to dynamic compliance, ME_BW_ – Mechanical energy

## **e-Table.4a. Multivariable logistic regression modelling analysis to determine the association MP_CRS_ adjusted for age, sex, oxygenation index, organ dysfunction score and type of ARDS.**

|  |  | **Estimate** | **Odds ratio** | **p-value** | **95% confidence interval** | |
| --- | --- | --- | --- | --- | --- | --- |
|  |  |  |  |  | **Upper** | **Lower** |
|  | **Model 1** | |  |  |  |  |
| AGE |  | 0.002 | 1.002 | 0.958 | 0.921 | 1.091 |
| SOFA at 24 hours |  | 0.263 | 1.300 | < .001 | 1.158 | 1.461 |
| OI at 24 hours |  | 0.046 | 1.047 | 0.174 | 0.980 | 1.120 |
| GENDER (M) |  | 0.582 | 1.789 | 0.112 | 0.873 | 3.669 |
| MP_CRS_ |  | 0.402 | 1.495 | 0.140 | 0.877 | 2.551 |
|  | **Model 2** | |  |  |  |  |
| AGE |  | -0.037 | 0.964 | 0.446 | 0.877 | 1.059 |
| SOFA at 24 hours |  | 0.240 | 1.272 | < .001 | 1.131 | 1.430 |
| OI at 24 hours |  | 0.065 | 1.067 | 0.068 | 0.995 | 1.145 |
| Type of ARDS (Indirect) |  | 0.944 | 2.571 | 0.027 | 1.114 | 5.933 |
| GENDER (M) |  | 0.641 | 1.899 | 0.087 | 0.910 | 3.961 |
| MP_CRS_ |  | 0.355 | 1.427 | 0.195 | 0.833 | 2.442 |

**e-Table.4b Multivariable logistic regression modelling analysis to determine the association MP_BW_ adjusted for age, sex, oxygenation index, organ dysfunction score and type of ARDS**

|  | **Estimate** | **Odds ratio** | **p-value** | **95% confidence interval** | |
| --- | --- | --- | --- | --- | --- |
|  |  |  |  | **Upper** | **Lower** |
| **Model 1** | |  |  |  |  |
| AGE | 0.023 | 1.023 | 0.618 | 0.936 | 1.119 |
| SOFA at 24 hours | 0.273 | 1.314 | < .001 | 1.172 | 1.473 |
| OI at 24 hours | 0.055 | 1.056 | 0.078 | 0.994 | 1.123 |
| GENDER (M) | 0.610 | 1.840 | 0.098 | 0.893 | 3.793 |
| MP_BW_ | 1.498 | 4.472 | 0.059 | 0.947 | 21.115 |
| **Model 2** | |  |  |  |  |
| AGE | -0.037 | 0.964 | 0.446 | 0.877 | 1.059 |
| SOFA at 24 hours | 0.240 | 1.272 | < .001 | 1.131 | 1.430 |
| OI at 24 hours | 0.065 | 1.067 | 0.068 | 0.995 | 1.145 |
| Type of ARDS (Indirect) | 0.944 | 2.571 | 0.027 | 1.114 | 5.933 |
| GENDER (M) | 0.641 | 1.899 | 0.087 | 0.910 | 3.961 |
| MP_BW_ | 0.355 | 1.427 | 0.195 | 0.833 | 2.442 |

## **e-Table.4c - Multivariable logistic regression modelling analysis to determine the association MP adjusted for age, sex, oxygenation index, organ dysfunction score and type of ARDS**

|  | **Estimate** | **Odds ratio** | **p-value** | **95% confidence interval** | |
| --- | --- | --- | --- | --- | --- |
|  |  |  |  | **Upper** | **Lower** |
| **Model 1** | |  |  |  |  |
| AGE | -0.107 | 0.899 | 0.124 | 0.784 | 1.030 |
| SOFA at 24 hours | 0.265 | 1.304 | < .001 | 1.161 | 1.463 |
| OI at 24 hours | 0.058 | 1.060 | 0.061 | 0.997 | 1.127 |
| GENDER (M) | 0.729 | 2.074 | 0.053 | 0.990 | 4.346 |
| MP | 0.097 | 1.102 | 0.063 | 0.995 | 1.221 |
| **Model 2** | |  |  |  |  |
| AGE | -0.117 | 0.890 | 0.095 | 0.776 | 1.021 |
| SOFA at 24 hours | 0.246 | 1.279 | < .001 | 1.139 | 1.437 |
| OI at 24 hours | 0.076 | 1.079 | 0.022 | 1.011 | 1.151 |
| Type of ARDS (Indirect) | 0.853 | 2.346 | 0.050 | 0.998 | 5.512 |
| GENDER (M) | 0.759 | 2.137 | 0.049 | 1.002 | 4.557 |
| MP | 0.073 | 1.076 | 0.164 | 0.971 | 1.192 |

**e-Table.4d Multivariable logistic regression modelling analysis to determine the association ME_BW_ adjusted for age, sex, oxygenation index, organ dysfunction score and type of ARDS**

|  | **Estimate** | **Odds ratio** | **p-value** | **95% confidence interval** | |
| --- | --- | --- | --- | --- | --- |
|  |  |  |  | **Upper** | **Lower** |
| **Model 1** | |  |  |  |  |
| AGE | -0.001 | 0.999 | 0.975 | 0.917 | 1.087 |
| SOFA at 24 hours | 0.274 | 1.315 | < .001 | 1.173 | 1.473 |
| OI at 24 hours | 0.052 | 1.053 | 0.099 | 0.990 | 1.119 |
| GENDER (M) | 0.611 | 1.842 | 0.099 | 0.892 | 3.803 |
| **ME_BW_** | **0.070** | **1.072** | **0.044** | **1.002** | **1.147** |
| **Model 2** | |  |  |  |  |
| AGE | -0.036 | 0.964 | 0.448 | 0.878 | 1.059 |
| SOFA at 24 hours | 0.254 | 1.289 | < .001 | 1.149 | 1.445 |
| OI at 24 hours | 0.069 | 1.071 | 0.037 | 1.004 | 1.143 |
| Type of ARDS (Indirect) | 0.885 | 2.424 | 0.039 | 1.046 | 5.616 |
| GENDER (M) | 0.672 | 1.957 | 0.076 | 0.932 | 4.108 |
| ME_BW_ | 0.060 | 1.061 | 0.085 | 0.992 | 1.136 |

OI – Oxygentaion Index, SOFA – sequential organ failure assessment score, MP – mechanical power, MP_BW_ – Mechanical power normalized to body weight, MP_CRS_ – Mechanical power normalized to dynamic compliance, ME_BW_ – Mechanical energy

1. **eTable 5a**

**Multivariable logistic regression modelling analysis to determine the association ME_BW_ adjusted for age, sex, oxygenation index, organ dysfunction score, type of ARDS AND MALNUTRITION.**

| **Coefficients** | | | | | | | | | | | | | | | | | | | |
| --- | --- | --- | --- | --- | --- | --- | --- | --- | --- | --- | --- | --- | --- | --- | --- | --- | --- | --- | --- |
|  | | | | | | | | | | **Wald Test** | | | | | | **95% Confidence interval  (odds ratio scale)** | | | |
|  | | **Estimate** | | **Standard Error** | | **Odds Ratio** | | **z** | | **Wald Statistic** | | **df** | | **p** | | **Lower bound** | | **Upper bound** | |
| (Intercept) |  | -4.899 |  | 0.803 |  | 0.007 |  | -6.105 |  | 37.271 |  | 1 |  | < .001 |  | 0.002 |  | 0.036 |  |
| AGE |  | -0.017 |  | 0.048 |  | 0.983 |  | -0.352 |  | 0.124 |  | 1 |  | 0.725 |  | 0.894 |  | 1.081 |  |
| SOFA24 |  | 0.249 |  | 0.059 |  | 1.282 |  | 4.181 |  | 17.484 |  | 1 |  | < .001 |  | 1.141 |  | 1.441 |  |
| OI24 |  | 0.066 |  | 0.033 |  | 1.068 |  | 1.978 |  | 3.912 |  | 1 |  | 0.048 |  | 1.001 |  | 1.140 |  |
| GENDER (M) |  | 0.535 |  | 0.387 |  | 1.707 |  | 1.381 |  | 1.907 |  | 1 |  | 0.167 |  | 0.799 |  | 3.647 |  |
| typeofards (indirect) |  | 1.009 |  | 0.436 |  | 2.743 |  | 2.313 |  | 5.348 |  | 1 |  | 0.021 |  | 1.166 |  | 6.451 |  |
| Malnutrition (Yes) |  | 0.795 |  | 0.416 |  | 2.215 |  | 1.909 |  | 3.644 |  | 1 |  | 0.056 |  | 0.979 |  | 5.010 |  |
| me24 |  | 0.077 |  | 0.042 |  | 1.080 |  | 1.839 |  | 3.382 |  | 1 |  | 0.066 |  | 0.995 |  | 1.172 |  |
|  | | | | | | | | | | | | | | | | | | | |
| *Note.*  SURVIVED1 level 'dead' coded as class 1. | | | | | | | | | | | | | | | | | | | |

| **Multicollinearity Diagnostics** | | | | | |
| --- | --- | --- | --- | --- | --- |
|  | | **Tolerance** | | **VIF** | |
| AGE |  | 0.808 |  | 1.237 |  |
| SOFA24 |  | 0.866 |  | 1.154 |  |
| OI24 |  | 0.762 |  | 1.313 |  |
| GENDER |  | 0.956 |  | 1.046 |  |
| typeofards |  | 0.773 |  | 1.293 |  |
| Malnutrition |  | 0.873 |  | 1.145 |  |
| me24 |  | 0.891 |  | 1.122 |  |
|  | | | | | |

**eTable 5b**

**Multivariable logistic regression modelling analysis to determine the association MP-24 hrs_,_ adjusted for age, sex, oxygenation index, organ dysfunction score, type of ARDS AND MALNUTRITION.**

| **Coefficients** | | | | | | | | | | | | | | | | | | | |
| --- | --- | --- | --- | --- | --- | --- | --- | --- | --- | --- | --- | --- | --- | --- | --- | --- | --- | --- | --- |
|  | | | | | | | | | | **Wald Test** | | | | | | **95% Confidence interval  (odds ratio scale)** | | | |
|  | | **Estimate** | | **Standard Error** | | **Odds Ratio** | | **z** | | **Wald Statistic** | | **df** | | **p** | | **Lower bound** | | **Upper bound** | |
| (Intercept) |  | -4.335 |  | 0.651 |  | 0.013 |  | -6.656 |  | 44.307 |  | 1 |  | < .001 |  | 0.004 |  | 0.047 |  |
| AGE |  | -0.125 |  | 0.068 |  | 0.882 |  | -1.837 |  | 3.373 |  | 1 |  | 0.066 |  | 0.772 |  | 1.008 |  |
| SOFA24 |  | 0.239 |  | 0.060 |  | 1.269 |  | 3.963 |  | 15.708 |  | 1 |  | < .001 |  | 1.128 |  | 1.428 |  |
| OI24 |  | 0.067 |  | 0.033 |  | 1.070 |  | 2.040 |  | 4.160 |  | 1 |  | 0.041 |  | 1.003 |  | 1.141 |  |
| GENDER (M) |  | 0.652 |  | 0.394 |  | 1.919 |  | 1.655 |  | 2.739 |  | 1 |  | 0.098 |  | 0.887 |  | 4.153 |  |
| typeofards (indirect) |  | 0.947 |  | 0.443 |  | 2.579 |  | 2.137 |  | 4.566 |  | 1 |  | 0.033 |  | 1.082 |  | 6.148 |  |
| Malnutrition (Yes) |  | 1.037 |  | 0.439 |  | 2.821 |  | 2.361 |  | 5.574 |  | 1 |  | 0.018 |  | 1.193 |  | 6.673 |  |
| MP24 |  | 0.146 |  | 0.073 |  | 1.157 |  | 1.993 |  | 3.973 |  | 1 |  | 0.046 |  | 1.002 |  | 1.335 |  |
|  | | | | | | | | | | | | | | | | | | | |
| *Note.*  SURVIVED1 level 'dead' coded as class 1. | | | | | | | | | | | | | | | | | | | |

| **Multicollinearity Diagnostics** | | | | | |
| --- | --- | --- | --- | --- | --- |
|  | | **Tolerance** | | **VIF** | |
| AGE |  | 0.434 |  | 2.303 |  |
| SOFA24 |  | 0.846 |  | 1.182 |  |
| OI24 |  | 0.760 |  | 1.316 |  |
| GENDER |  | 0.933 |  | 1.072 |  |
| typeofards |  | 0.763 |  | 1.311 |  |
| Malnutrition |  | 0.780 |  | 1.282 |  |
| MP24 |  | 0.353 |  | 2.830 |  |
|  | | | | | |

**eTable 5c**

**Multivariable logistic regression modelling analysis was used to determine the association MPBW at 24 hrs, adjusted for age, sex, oxygenation index, organ dysfunction score, type of ARDS, and MALNUTRITION.**

| **Coefficients** | | | | | | | | | | | | | | | | | | | |
| --- | --- | --- | --- | --- | --- | --- | --- | --- | --- | --- | --- | --- | --- | --- | --- | --- | --- | --- | --- |
|  | | | | | | | | | | **Wald Test** | | | | | | **95% Confidence interval  (odds ratio scale)** | | | |
|  | | **Estimate** | | **Standard Error** | | **Odds Ratio** | | **z** | | **Wald Statistic** | | **df** | | **p** | | **Lower bound** | | **Upper bound** | |
| (Intercept) |  | -4.672 |  | 0.746 |  | 0.009 |  | -6.265 |  | 39.252 |  | 1 |  | < .001 |  | 0.002 |  | 0.040 |  |
| AGE |  | -0.001 |  | 0.051 |  | 0.999 |  | -0.019 |  | 3.465×10^-4^ |  | 1 |  | 0.985 |  | 0.904 |  | 1.104 |  |
| SOFA24 |  | 0.248 |  | 0.059 |  | 1.281 |  | 4.162 |  | 17.320 |  | 1 |  | < .001 |  | 1.140 |  | 1.439 |  |
| OI24 |  | 0.071 |  | 0.033 |  | 1.073 |  | 2.145 |  | 4.600 |  | 1 |  | 0.032 |  | 1.006 |  | 1.145 |  |
| MPBW 24 |  | 1.628 |  | 0.963 |  | 5.095 |  | 1.691 |  | 2.860 |  | 1 |  | 0.091 |  | 0.772 |  | 33.623 |  |
| GENDER (M) |  | 0.546 |  | 0.386 |  | 1.726 |  | 1.413 |  | 1.997 |  | 1 |  | 0.158 |  | 0.810 |  | 3.678 |  |
| typeofards (indirect) |  | 1.044 |  | 0.438 |  | 2.841 |  | 2.385 |  | 5.687 |  | 1 |  | 0.017 |  | 1.204 |  | 6.701 |  |
| Malnutrition (Yes) |  | 0.767 |  | 0.417 |  | 2.152 |  | 1.840 |  | 3.386 |  | 1 |  | 0.066 |  | 0.951 |  | 4.870 |  |
|  | | | | | | | | | | | | | | | | | | | |
| *Note.*  SURVIVED1 level 'dead' coded as class 1. | | | | | | | | | | | | | | | | | | | |

| **Multicollinearity Diagnostics** | | | | | |
| --- | --- | --- | --- | --- | --- |
|  | | **Tolerance** | | **VIF** | |
| AGE |  | 0.726 |  | 1.378 |  |
| SOFA24 |  | 0.867 |  | 1.154 |  |
| OI24 |  | 0.785 |  | 1.273 |  |
| MPBW 24 |  | 0.838 |  | 1.193 |  |
| GENDER |  | 0.956 |  | 1.046 |  |
| typeofards |  | 0.764 |  | 1.308 |  |
| Malnutrition |  | 0.872 |  | 1.146 |  |
|  | | | | | |

**eTable 5d**

**Multivariable logistic regression modelling analysis to determine the association MP_CRS_-24 hrs_,_ adjusted for age, sex, oxygenation index, organ dysfunction score, type of ARDS AND MALNUTRITION.**

| **Coefficients** | | | | | | | | | | | | | | | | | | | |
| --- | --- | --- | --- | --- | --- | --- | --- | --- | --- | --- | --- | --- | --- | --- | --- | --- | --- | --- | --- |
|  | | | | | | | | | | **Wald Test** | | | | | | **95% Confidence interval  (odds ratio scale)** | | | |
|  | | **Estimate** | | **Standard Error** | | **Odds Ratio** | | **z** | | **Wald Statistic** | | **df** | | **p** | | **Lower bound** | | **Upper bound** | |
| (Intercept) |  | -4.211 |  | 0.634 |  | 0.015 |  | -6.639 |  | 44.071 |  | 1 |  | < .001 |  | 0.004 |  | 0.051 |  |
| AGE |  | -0.016 |  | 0.049 |  | 0.984 |  | -0.324 |  | 0.105 |  | 1 |  | 0.746 |  | 0.894 |  | 1.083 |  |
| SOFA24 |  | 0.234 |  | 0.061 |  | 1.264 |  | 3.856 |  | 14.872 |  | 1 |  | < .001 |  | 1.122 |  | 1.423 |  |
| OI24 |  | 0.061 |  | 0.035 |  | 1.063 |  | 1.727 |  | 2.983 |  | 1 |  | 0.084 |  | 0.992 |  | 1.139 |  |
| GENDER (M) |  | 0.493 |  | 0.385 |  | 1.637 |  | 1.282 |  | 1.643 |  | 1 |  | 0.200 |  | 0.770 |  | 3.480 |  |
| typeofards (indirect) |  | 1.074 |  | 0.437 |  | 2.927 |  | 2.461 |  | 6.055 |  | 1 |  | 0.014 |  | 1.244 |  | 6.887 |  |
| Malnutrition (Yes) |  | 0.859 |  | 0.423 |  | 2.360 |  | 2.028 |  | 4.112 |  | 1 |  | 0.043 |  | 1.029 |  | 5.411 |  |
| MPCRS |  | 0.534 |  | 0.342 |  | 1.705 |  | 1.562 |  | 2.439 |  | 1 |  | 0.118 |  | 0.873 |  | 3.331 |  |
|  | | | | | | | | | | | | | | | | | | | |
| *Note.*  SURVIVED1 level 'dead' coded as class 1. | | | | | | | | | | | | | | | | | | | |

| **Multicollinearity Diagnostics** | | | | | |
| --- | --- | --- | --- | --- | --- |
|  | | **Tolerance** | | **VIF** | |
| AGE |  | 0.787 |  | 1.270 |  |
| SOFA24 |  | 0.845 |  | 1.184 |  |
| OI24 |  | 0.682 |  | 1.467 |  |
| GENDER |  | 0.959 |  | 1.043 |  |
| typeofards |  | 0.765 |  | 1.308 |  |
| Malnutrition |  | 0.844 |  | 1.185 |  |
| MPCRS |  | 0.714 |  | 1.400 |  |
|  | | | | | |

1. **e-Table 6:**  Correlations of 28-day ventilator-free days with mechanical power, normalized mechanical power, mechanical energy and mechanical power normalized to compliance.

| Correlation of 28-day ventilator free days | Spearman’s correlation coefficient (rho) | p-value |
| --- | --- | --- |
| Mechanical power (MP)  1– 4 hours  24 hours | -0.130  -0.187 | 0.190  0.059 |
| Normalized Mechanical power (MP_BW_)  1 – 4 hours  24 hours | -0.117  -0.212 | 0.239  0.032 |
| Mechanical Energy (ME_BW_) 1 – 4 hours  24 hours | -0.018  -0.277 | 0.806  <0.0001 |
| Mechanical power normalized to respiratory system compliance (MP_CRS_)  1 – 4 hours  24 hours | -0.268  -0.471 | <0.0001  <0.0001 |

##

1. **eFigure-1: Figure showing probability of mortality at day-28 with mechanical energy at 24 hours**

**
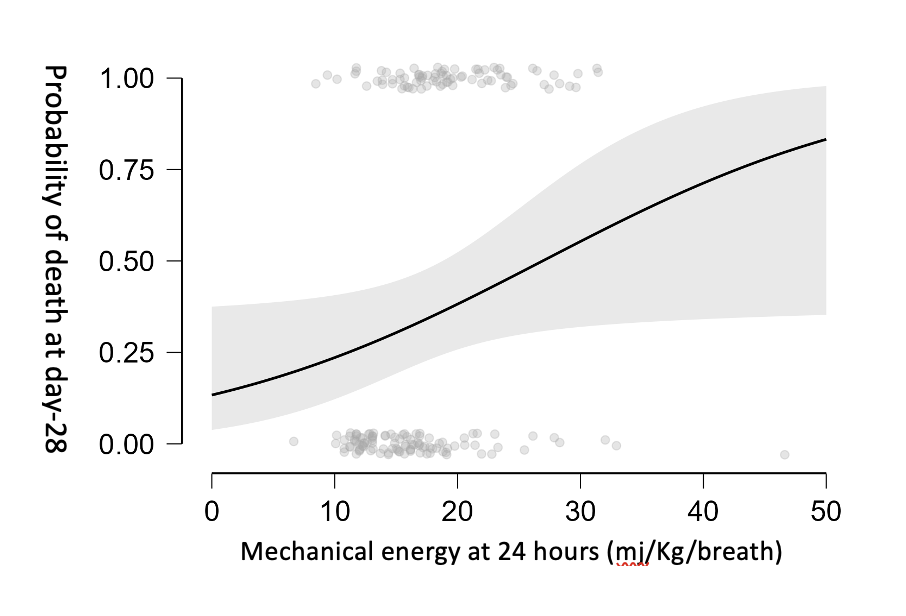
**
